# Comparison of Graphites Intercalated with Fluorine as Slow Neutron Reflectors

**DOI:** 10.3390/ma17235972

**Published:** 2024-12-06

**Authors:** Batiste Clavier, Valentin Czamler, Marc Dubois, Killian Henry, Valery Nesvizhevsky, Elodie Petit

**Affiliations:** 1 Clermont Auvergne INP, Université Clermont Auvergne, 63178 Aubière, France; batiste.clavier@uca.fr (B.C.); killian.henry@univ-lorraine.fr (K.H.); elodie.petit@uca.fr (E.P.); 2 NPP/DS, Institut Max von Laue—Paul Langevin, 38042 Grenoble, France

**Keywords:** neutron reflector, graphites intercalated with fluorine, bragg diffraction

## Abstract

The use of neutron reflectors is an effective method for improving the quality of neutron sources and neutron delivery systems. In this work, we further develop the method based on the Bragg scattering of neutrons in crystals with large interplanar distances. We compare samples of differently prepared fluorine intercalated graphites by measuring the total cross section for the interaction of neutrons with the samples, depending on the neutron wavelength. The Brag scattering cross section is expected to be the dominant part of the total cross section in all the cases. The results show that all samples provide high reflection efficiency over the entire range of the so-called “neutron reflectivity gap” and beyond it, and that they also allow for the choosing of the optimal intercalation methods.

## 1. Introduction

The development of neutron reflectors [1,2,3,4,5] with high albedo [6,7] over a wide range of wavelengths $R(\lambda_{n})$ is an effective method for increasing neutron fluxes and improving the quality of neutron sources [8,9], which is of great interest for both fundamental and applied research [10,11,12,13,14,15,16,17].

For the neutron reflectors considered in this work, the $\lambda_{n}$ ranges are of interest, which can be divided roughly as follows: thermal neutrons (TN) (0.05 nm $<\lambda_{TN}<0.4$ nm), cold neutrons (CN) (0.4 nm $<\lambda_{CN}<2.0$ nm), very cold neutrons (VCN) (2.0 nm $<\lambda_{VCN}<50.0$ nm), and ultracold neutrons (UCN) (50.0 nm $<\lambda_{UCN}$).

The development of neutron reflectors is especially interesting for those $\lambda_{n}$ ranges for which alternative methods had been previously absent or inefficient. In particular, the so-called “neutron reflectivity gap” had existed until recently in the hard part of the VCN range and in the soft part of the CN range (0.7 nm $<\lambda_{n}<10.0$ nm). To overcome this limitation, we have developed two methods.

The first method is based on the huge coherent enhancement of VCN scattering on nanoparticles. Nanodiamonds (NDs) [18,19,20,21,22,23,24,25,26,27,28,29] are the best type of such nanoparticles. This method has been thoroughly investigated in a large number of works [30,31,32,33,34,35,36,37,38,39,40,41,42]. The best implementation of this method so far is fluorinated detonation nanodiamond (F-DND) powders [43]. They provide record albedo in a wide range of wavelengths (1.0 nm $<\lambda_{n}<50.0$ nm); however, with a decrease in $\lambda_{n}$, the required reflector thickness rapidly increases and reaches values that are difficult to implement in practice. Therefore, for practically acceptable reflector thicknesses, the effective range of F-DND reflectors is better defined as (2.0 nm $<\lambda_{n}^{F-DND}<50.0$ nm) which coincides precisely with the VCN range.

The second method is based on the Bragg–Wulf scattering [44,45] of neutrons by crystals with large interplane distances; here, crystals with standard interplane distances are not efficient [46,47]. The best implementation of this method, to date, is in graphite powders intercalated with fluorine [48,49]. They provide record albedo and acceptable reflector thickness, at least in the wavelength range (0.7 nm $<\lambda_{n}<2.0$ nm). The advantages of materials of this type are the presence of only carbon and fluorine atoms, which have small neutron capture cross sections, large interplanar distances up to ∼9 Å, high thermal and chemical compound stability as well as hydrophobic characteristics.

In the case of an isotropic scatterer, the angular distribution of neutrons reflected from the reflector plane is close to isotropic in the corresponding angular hemisphere [48,50,51]. In the case of an anisotropic scatterer [49,52,53,54], a large increase in the efficiency of the reflection of neutrons that are incident on the surface of the scatterer at certain angles is possible. However, this increase is accompanied by a large weakening of the efficiency of reflection at other angles. The choice of reflector is carried out in accordance with the type and parameters of the task. In particular, the isotropic scattering of neutrons allows them to be effectively stored in closed volumes and/or extracted preferentially in certain directions [55].

In ref. [48], a dedicated home-made neutron diffractometer was built for measurements of double-differential neutron cross sections of crystals with specific large interlayer distances. The advantage of this method is the possibility of the direct and absolute measurement of neutron diffraction cross sections in a wide range of $\lambda_{n}$ and scattering angles θ. However, such a measurement requires the construction of this experimental configuration for each such measurement at the PF1B instrument at the ILL, as well as a relatively long time for collecting statistics.

Therefore, we carried out a series of short measurements of neutron transmission through various samples of intercalated graphite with the following goals: to compare the efficiency of neutron scattering depending on the intercalation method and the size of intercalated graphite crystals, to expand the wavelength range of measurements towards larger values, and to select the types of samples with which in the next stage of full measurements of the double-differential scattering cross section will be performed, similar to those presented in ref. [48].

## 2. Materials and Methods

### 2.1. Samples

The following types of graphite fluoride powders were investigated: commercial samples were obtained from Advance Research Chemicals, Inc. (ARC) (Catoosa, OK, USA), denoted as ARC series samples, and synthesized at the Institute of Chemistry of Clermont-Ferrand in order to cover different ranges of properties, i.e., chemical composition (F/C molar ratio), structural type, granulometry and C-F bonding. Most of the samples exhibit covalency for the C-F bonds due to their synthesis with pure elemental fluorine (F_2_) at high temperatures, in the 350–600 °C range. The details of the fluorination of graphite are given in the corresponding papers for CF_0.51_ and CF_0.61_ [56]. CF_0.71_ was synthesized with the sample graphite but the temperature was fixed at 420 °C in order to increase the fluorine content; x in CF_*x*_, x is equal to the molar ratio. These 3 samples constitute the second series. Recently, we focus our efforts to prepare graphite fluorides with a high content of the ${\left(C_{2}F\right)}_{n}$ phase [48]. FKS450 and FNG series (FNG440, FNG460, and FNG600) were then obtained. Only one sample was prepared at room temperature using a catalytic gaseous mixture F_2_, HF, and IF_5_ [57]. The sample is denoted RT-GF.

The sample parameters are summarized in Table 1. Data are provided by the ARC provider for the commercial products. For the other graphite fluoride samples, the F/C ratio and the ${\left(C_{2}F\right)}_{n}$ fraction are extracted from the solid-state nuclear magnetic resonance (see ref. [48]). The granulometry reported is the one of the starting graphite considering that the value does not change during the fluorination. The interlayer distances are extracted from X-ray diffraction [48,57].

The studied samples can be divided into several groups, allowing us to study the efficiency of reflectors depending on the following parameters, Pi:

P1. The relative content of carbon and fluorine (the ARC series) with the same ${\left(CF\right)}_{n}$ structural type and granulometry;

P2. The fluorine content with the same ${\left(C_{2}F\right)}_{n}$ structural type and granulometry (FNG440, FNG460, and FNG600);

P3. The crystal size (CF_0.61_, FKS450, FNG440) with a high content of the ${\left(C_{2}F\right)}_{n}$ phase;

P4. The relative content of non-fluorinated graphite (CF_0.51_, CF_0.61_, and CF_0.71_) with the same granulometry;

P5. The C-F bonding, i.e., the weakened covalence for RT-GF and the covalence for CF_0.71_, with the same fluorine content and granulometry.

The characterization of the samples will show their main characteristics that are useful for the present work. If the readers need further details, see ref. [48]. The granulometry reported is the one of the starting graphite considering that the value does not change during the fluorination. The interlayer distances are extracted from X-ray diffraction [48,56,57].

### 2.2. FTIR Measurements

Fourier-transform infrared (FTIR) spectra were recorded on a Nicolet 6700 FT-IR (Thermo scientific) spectrometer in the transmission mode at 4 cm^−1^ resolution, and 256 scans were taken for each spectrum. NMR experiments were carried out with a Bruker Avance spectrometer, with the working frequencies for ^13^C and ^19^F equal to 73.4 and 282.2 MHz, respectively. A Magic Angle Spinning (MAS) probe (Bruker) operating with 2.5 mm rotors was used. For MAS spectra, a simple sequence was performed with a single π/2 pulse length of 4.0 and 3.5 µs for ^19^F and ^13^C, respectively. For MAS measurements, the samples must be fragmented. ^13^C chemical shifts were externally referenced to tetramethylsilane (TMS), while ^19^F chemical shifts were referenced with respect to CFCl_3_. X-ray diffraction (XRD) patterns were recorded using a PANalytical X’PERT X-ray reflection diffractometer with Cu (K*α*) radiation ($\lambda_{K_{\alpha 1}}=1.5406$ Å).

### 2.3. Characterization and Preparation of the Graphite Fluorides

**Figure 1:**
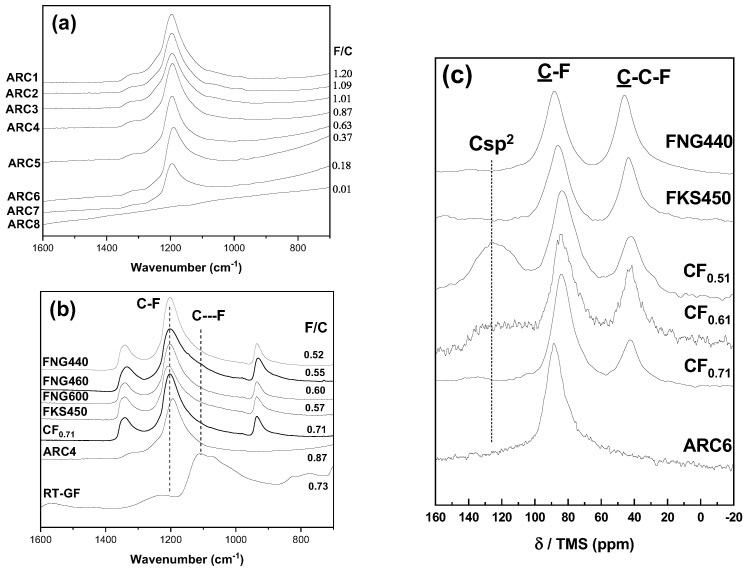
Infrared spectra (ATR mode) of the ARC series (**a**) and of the other graphite fluoride samples (**b**); ^13^C MAS NMR spectra of selected samples (with the spinning rate of 10 KHz) (**c**).

The first characteristic studied is the fluorine content with the same structure ((CF)_*n*_ as that revealed by the interlayer distance of 6.2 Å, see Table 1), C-F bonding, and granulometry. In order to evidence those properties, FTIR spectra of the ARC series were recorded (Figure 1). The vibration band is observed at around 1200 cm^−1^ for all the samples. This wavenumber is typical of covalent C-F bonds [58,59]. In accordance with the XRD data (the interlayer distance of 6.2 Å, typical of the (CF)_*n*_ structural type), the spectra of ARC samples do not exhibit vibrational bands at 1350 and 940 cm^−1^ which are typical of graphite fluoride with a high content of ${\left(C_{2}F\right)}_{n}$ phase. Only the F/C ratio changes in the ARC series (Parameter P1).

The vibrational bands of the ${\left(C_{2}F\right)}_{n}$ phase at 1350 and 940 cm^−1^ are observed for the FNG series whatever the fluorination temperature and the fluorine content are (Figure 1b). XRD data evidence the interlayer distance of 9.2 Å which is typical of the ${\left(C_{2}F\right)}_{n}$ structural type. The ^13^C NMR spectra of the FNG series are composed of two main lines at 84 and 42 ppm which are assigned to the carbon atoms involved in the covalent C-F bonds (denoted as C-F in Figure 1c) and non-fluorinated carbons with sp^3^ hybridization (C-C-F) [56,57,59,60]. C-C-F are present only in the ${\left(C_{2}F\right)}_{n}$ phase. According to the integrated surface of the lines S_*C*-*F*_ and S_*C*-*C*-*F*_, the ratio $100\;\cdot \;{\left(C_{2}F\right)}_{n}/{\left(CF\right)}_{n}=$100· S_*C*-*C*-*F*_/S_*C*-*F*_ gives the percentage of the ${\left(C_{2}F\right)}_{n}$ structural type. The data are summarized in Table 1. The ${\left(C_{2}F\right)}_{n}$ fractions are 94, 96, and 67% for FNG440, FNG460, and FNG600, respectively. This series will be used to investigate the effect of the F/C ratio for samples with the ${\left(C_{2}F\right)}_{n}$ structural type (P2).

The samples CF_0.61_ and FKS450 have the same characteristics as FNG440 but with a different granulometry. These 3 samples allow the crystal size to be investigated (P3).

Because of the presence of sp^2^ carbon atoms, the FTIR spectra of CF_0.51_ and CF_0.61_ are ill-defined and ^13^C MAS NMR spectra are shown to have the main information, in particular to evidence the graphitic region (Figure 1c). Non-fluorinated carbon atoms, with sp^2^ hybridization, result in a resonance line in the 120–135 ppm range [56,57,59,60]. The shift in comparison with graphite (120 ppm) is explained by the interaction with C-F bonds in their neighboring. The intensity of the line at 130 ppm decrease with the F/C ratio in the CF_0.51_, CF_0.61_, and CF_0.71_ series which can be used to study the effect of the residual graphitic region (P4).

RT-GF is the only sample with a vibration band of a wavenumber that is different than 1200 cm^−1^, i.e., 1100 cm^−1^, which is related to weakened covalent C-F bonds (C-F in Figure 1b, covalent C-F is denoted as C-F). The fluorination of graphite at room temperature results in such C-F bonding. RT-GF will be compared with a sample which exhibits the same fluorine content and closely the same fluorine content, i.e., CF_0.71_, in order to underline the effect of C-F bonding (P5).

### 2.4. Neutron Measurements

The PF1B instrument at ILL has several unique features that facilitate the study of neutron reflectors based on the Bragg scattering of neutrons by crystals with large interplanar distances. These include a wide range of neutron wavelengths and a record neutron density in the phase space (this is important for covering the range of neutron wavelengths corresponding to the reflectivity gap), the ability to completely change the configuration of this instrument and optimize it for the scientific task at hand, low backgrounds necessary for studies with long-wavelength neutrons, and a sufficiently large experimental area, allowing us to, in particular, install a diffractometer using a position-sensitive detector that can be installed in the direction of nearly any scattering angle. Such a specialized diffractometer was used in the work [48] for our previous studies of neutron diffraction in intercalated graphites.

**Figure 2:**
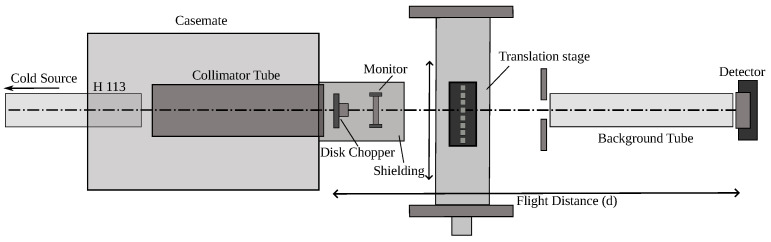
Outline of the experimental setup of the PF1B instrument. The cold neutron beam enters from the ballistic m=2 supermirror ballistic guide H113 on the left and passes through the collimation system containing a series of decreasing apertures in a vacuum. When entering the experimental zone, the beam passes through a disc chopper followed by a monitor and the sample. An m=3 supermirror behind the sample provides a cut-off wavelength of about 5*Å* and allows us to significantly reduce the background in the thermal neutron range due to a non-negligible transmission through the chopper disk. The detector was a ^3^He-counter with almost 100% efficiency. A translation stage allows us to scan through up to 8 samples per run.

For neutron measurements, the following experimental configuration was installed: (1) the primary collimator made it possible to reduce backgrounds in the experimental area, (2) the chopper made it possible to measure the neutron flux depending on the value of $\lambda_{n}$, (3) the cassette had samples that could be moved automatically or installed manually, (4) the reflection of the neutron beam from the supermirror made it possible to remove the hard part of the neutron spectrum and, thus, avoid the overloading of (5) the neutron detector, and (6) to further reduce the background, the detector was protected from all sides, except for the direction of the propagation of the neutron beam (see Figure 2).

Graphite fluoride powders were poured into cylindrical holders with thin aluminum walls that were 0.2 mm thick. The diameters of half of the holders were 0.8 cm, and those of the other half were 1.2 cm. The neutron spectrum incident on the detector was measured with an empty aluminum holder. The probability of beam attenuation by samples was measured as a function of $\lambda_{n}$.

#### 2.4.1. Justification for the Choice of Experimental Parameters in Neutron Measurements

The sample thickness was chosen in such a way that, on the one hand, the attenuation of the neutron beam was small (∼10^−1^) and the probability of multiple neutron scatterings in the samples did not exceed a few percent, and, on the other hand, it was large enough for the difference in neutron fluxes without/with a sample to be measured with the required statistical accuracy in a reasonable amount of time. Note that the intensity of the incident neutron beam decreases rapidly with increasing $\lambda_{n}$, so the statistical accuracy decreases rapidly with increasing $\lambda_{n}$. To increase the maximum $\lambda_{n}$ value corresponding to statistically significant attenuation factors, significant time is required for collecting statistics. Fortunately, as we will see below from the analysis of the results obtained, the maximum value of $\lambda_{n}$ approximately corresponds to the upper value of the interval of interest to us.

Total cross section $\sigma_{tot}\left(\lambda_{n}\right)$ for the elimination of neutrons from the direct beam consists of absorption cross section $\sigma_{abs}\left(\lambda_{n}\right)$ and scattering cross section $\sigma_{sc}\left(\lambda_{n}\right)$.

(1) $$\sigma_{tot}\left(\lambda_{n}\right)=\sigma_{abs}\left(\lambda_{n}\right)+\sigma_{sc}\left(\lambda_{n}\right).$$

In turn, the scattering cross section can be divided into several components in our specific conditions: the diffraction cross section on the crystal lattice $\sigma_{diff.cryst}\left(\lambda_{n}\right)$, the small-angle scattering cross section on density inhomogeneities $\sigma_{diff.SANS}\left(\lambda_{n}\right)$, and the incoherent cross section $\sigma_{inc}\left(\lambda_{n}\right)$:

(2) $$\sigma_{sc}\left(\lambda_{n}\right)=\sigma_{diff.cryst}\left(\lambda_{n}\right)+\sigma_{diff.SANS}\left(\lambda_{n}\right)+\sigma_{inc}\left(\lambda_{n}\right).$$

All cross sections are averaged over all angles. The characteristic values of $\sigma_{diff.cryst}\left(\lambda_{n}\right)$ for the samples of graphite fluoride powders are equal to several barns per atom in the range of interest to us ∼0.5 nm $<\lambda_{n}<2.0$ nm as follows from the direct measurements [48]. Compared to $\sigma_{diff.cryst}\left(\lambda_{n}\right)$, the neutron capture cross section $\sigma_{abs}\left(\lambda_{n}\right)$ is negligible, since the capture cross sections for carbon and fluorine atoms are small ($\sigma_{abs}^{C}=3.5$ mb and $\sigma_{abs}^{F}=9.6$ mb, at the thermal neutron wavelength) and the impurities in the samples are insignificant. In order to reduce the contribution of $\sigma_{diff.SANS}\left(\lambda_{n}\right)$, we used samples with sufficiently large granules (7.5–400 µm, a characteristic SANS angle is <3 $\;\cdot \;10^{-4}$) and the angular acceptance of the detector was sufficiently large (2 ·$10^{-2}$). Moreover, we verified experimentally that placing the sample closer to the detector and moving it from the position upstream from the supermirror to the position downstream from the supermirror does not lead to a significant change in the result in the range $\lambda_{n}$ of interest. $\sigma_{inc}\left(\lambda_{n}\right)$ values for the particular types of crystals are not known but their typical values are significantly smaller than $\sigma_{diff.cryst}\left(\lambda_{n}\right)$ as follows from their composition and our direct preceding measurements [48].

That is, we have reason to believe that measuring $\sigma_{tot}\left(\lambda_{n}\right)$ is a good way to estimate $\sigma_{diff.cryst}\left(\lambda_{n}\right)$ for the particular samples and conditions used. However, we do not claim to accurately measure $\sigma_{diff.cryst}\left(\lambda_{n}\right)$ using the neutron transmission method. Nevertheless, the trends emerging from comparisons of different analogous samples with a gradually changing parameter are reliable.

#### 2.4.2. Neutron Spectrum Shaping and Wavelength Calibration

**Figure 3:**
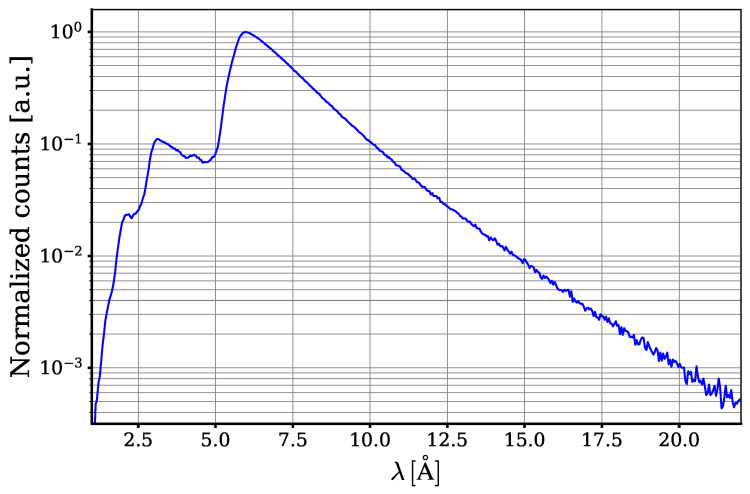
Neutron spectrum measured without a sample. Intensity on the vertical axis is in arbitrary units. Neutron wavelength on the horizontal axis is in Å.

The measured spectrum of neutrons without a sample and after the m = 3 spectrum-shaping supermirror is shown in Figure 3. The decrease in intensity for neutron wavelengths less than ∼6 Å is due to the passage of neutrons through the m = 3 supermirror and their subsequent absorption. The decrease in intensity for neutron wavelengths greater than ∼6 Å is due to their deficiency in the initial neutron beam, as well as their suppression due to the angular collimation of the neutron beam. The spectrum is specially shaped to cover the range of interest and reduce the background.

To calibrate the wavelength scale of the experimental setup, the well-known Bragg edges of aluminium at λ=4.045Å and λ=4.675Å were used. To this extent, the transmission of a 2.125 cm aluminium cylinder was measured. The calculated cross section is used to calibrate the wavelength scale.

## 3. Results

**Figure 4:**
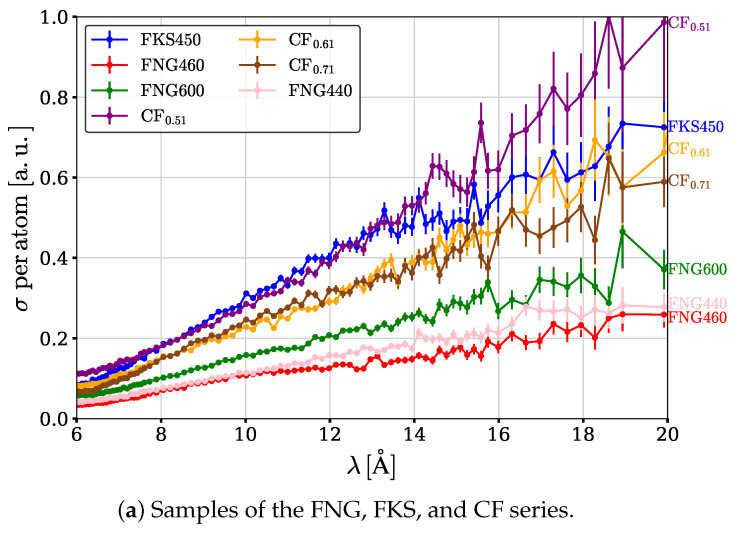

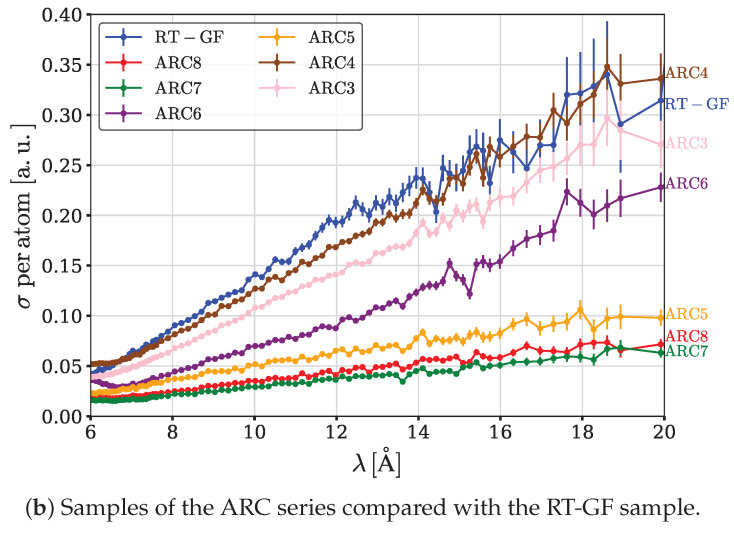
Total cross sections of interactions of neutrons (σ) with different intercated graphite samples, per atom in arbitrary units. The normalization per atom uses the mass density and chemical composition presented in Table 1. The results are presented in two figures to improve visibility.

Total cross sections of neutron interaction with intercalated graphite samples are shown in Figure 4a,b.

### 3.1. Total Cross Section Versus Fluorine Content for ${\left(CF\right)}_{n}$

The comparison of results for different samples from the ARC series (see Table 1) allows us to assess the impact of the relative contents of carbon and fluorine. Grain size is the same: 1–45 µm. Inter-plane distance is the same: 6.2 Å. Structural type is the same: ${\left(CF\right)}_{n}$. The F/C ratio varies from 1.01 (ARC3) to 0.01 (ARC8). Cross sections increase consistently with the increasing F/C ratio in the studied range (0.01–1.01).

### 3.2. Total Cross Section Versus Fluorine Content for ${\left(C_{2}F\right)}_{n}$

Comparing the samples of FNG440, FNG460, and FNG600 allows us to assess the impact of the fluorine content. The structural type is the same: ($C_{2}{F)}_{n}$. The granulometry is the same: 200–400 µm. Inter-plane distances are the same: 9.2 Å. The ($C_{2}{F)}_{n}$ varies from 95% (FNG440, FNG460, i.e., 5% of the (CF)_*n*_ phase) to 67% (FNG600, 33% of the (CF)_*n*_ phase). Cross sections decrease with increasing amount of content in the (CF)_*n*_ phase in the studied range (5–33%).

### 3.3. Total Cross Section Versus Crystal Size

Comparing the samples of CF_0.61_, FKS450, and FNG440 allows us to assess the impact of crystal size. In all these samples, the content of ${\left(C_{2}F\right)}_{2}$ is high. No net tendency at shorter wavlengths is observed in the range (7.5 µm for CF_0.61_, 44 µm for FKS450, while a drop in efficiency is present for larger sizes: 200–400 µm for FNG440). The later is apparently due to the screening of Bragg scattering in “too large” crystal sizes. The point is that if the crystal size is larger than the characteristic extinction length, then only a layer of the crystal of such thickness effectively participates in neutron diffraction, and deeper layers of the crystal do not contribute to diffraction. Another manifestation of the screening effect is the suppression of cross sections at about 13 Å for FKS450 compared to CF_0.51_. This observation allows us to shift the “optimum” crystal size, in the linear approximation, from 44 µm (FKS450) to ∼30 µm.

### 3.4. Total Cross Section Versus Content of Non-Fluorinated Graphite

Comparing the samples of CF_0.51_, CF_0.61_, and CF_0.71_ allows us to assess the impact of the relative content of non-fluorinated graphite. The composition can be expressed as C_0.49_(CF)_0.51_, C_0.39_(CF)_0.61_, and C_0.29_(CF)_0.71_, respectively. Grain sizes are the same, i.e., 7.5 µm. High graphitic phase content corresponds to noticeably better performance in the studied range (0.29–0.49). This might be a result of the combination of two efficient types of scattering: in graphite at shorter neutron wavelengths and in intercalated graphite at longer neutron wavelengths.

### 3.5. Total Cross Section Versus C-F Bonding

Comparing the samples of CF_0.71_ and RT-GF allows us to assess the impact of C-F bonding, i.e., the weakened covalence for RT-GF and the covalence for CF_0.71_. Fluorine content is the same, and grain sizes are the same. The CF_0.71_ sample with covalent C-F bonds shows a better performance than the RT-GF sample with weakened covalence.

### 3.6. Wavelength Dependence of Total Cross Sections

For all samples studied, the total interaction in cross sections increases over the entire range of the wavelengths of interest (6–20 Å, Figure 4). This observation is in good agreement with the conclusions of the paper [48], in which this range of neutron wavelengths, however, was not investigated experimentally at the largest wavelengths. The increase in the diffraction cross section with increasing neutron wavelength (within the experimentally studied range of the reflectivity gap) is explained by the fact that scattering is most effective at wavelengths of the order of interplanar distances. It is expected that for crystals with identical interplanar distances, the cross sections are high up to values equal to two interplanar distances. However, it should be remembered that all the studied samples consist of a certain set of interplanar distances, and therefore the tendency may persist for somewhat larger wavelengths. The largest cross section values, as well as the fastest growth of cross sections with increasing neutron wavelengths, are observed for samples with the largest interplanar distances.

## 4. Discussion

As noted in the introduction, we performed a series of measurements of the probability of transmitting a neutron beam through various samples of fluorine-intercalated graphite. The wavelength range of the formed neutron beam completely covered the range of the so-called gap in the effective reflectivity of neutron reflectors. For the samples chosen and the measurement parameters, the main part of the total cross section was the diffraction cross section on the crystal lattice.

The properties of the samples were chosen in such a way as to allow an experimental study of the cross section values depending on several important parameters (neutron wavelength, crystal size in the powder, intercalation type, fluorine content, non-fluorinated graphite content, C-F bonds, etc.). Theoretical study of these cross sections is practically impossible, since it involves precise knowledge of the structure of each sample and requires a fairly complex theoretical calculation. Direct experimental measurement of the double-differential neutron diffraction cross section on all samples in the required range of neutron wavelengths is also unrealistic due to the fact that it requires the construction of a specialized diffractometer (for example, as described in the work [48]), as well as a very long measurement (due to a major decrease in statistics for large neutron wavelengths).

Therefore, the strategy we have chosen seems to be optimal and the only possible one: (1) draw qualitative conclusions about the properties of the samples based on measurements of the total cross sections, then (2) prepare a few samples, which seem optimal to us based on these conclusions, and (3) quantitatively measure the diffraction cross sections using a specialized diffractometer [48]. The main conclusions are given below.

## 5. Conclusions

We have studied for the first time the total cross sections of neutron interactions with different samples of intercalated graphite in the entire range of wavelengths covering the so-called reflectivity gap. Since the main part of the total cross section under the specific experimental conditions is the neutron diffraction cross section on the crystal lattice, and also because we compared samples that differ in one parameter (i.e., the fluorination rate with the (CF)_*n*_ structural type, the relative content of the residual graphitic phase, the relative content of the (C_2_F)_*n*_ phase, C-F bonding, and crystal size), we believe that the qualitative conclusions about the behavior of the diffraction cross sections are sufficiently reliable. In particular, we have experimentally confirmed for the first time the increase in diffraction cross sections with increasing neutron wavelengths in the entire range corresponding to the reflectivity gap, including the part of this range that was inaccessible to the measurements [48]. Particularly rapid growth and large values are observed for samples with large interplanar distances (high contents of the (C_2_F)_*n*_ phase). An optimum type of intercalation corresponds approximately to the CF_0.51_ sample. No net impact of crystal size on the cross sections for shorter wavelengths is observed in the range of 7.5–44 µm, while it drops down at the crystal size of 200–400 µm, due to the screening of Bragg scattering by “too big” crystals. A relative suppression of cross sections above 13 Å for the FKS450 sample allows us to estimate, in the linear approximation, that the suppression would vanish for the entire range of the wavelength interest at the crystal size of ∼30 µm. As larger grain sizes allow for a larger volume density, our current “optimum” reflector should consists of ∼30 µm CF_0.51_ powder with covalent bonds, or around these parameters. The (C_2_F)_*n*_ phase is an intermediate compound which can be described as a stage 2 covalent F-GICs. Its synthesis with molecular fluorine F_2_ results often in the coexistence with either a residual graphitic phase or a (CF)_*n*_ phase (the presence of both is possible), if the fluorination duration is insufficient and/or the temperature is not optimal. Typical cases in our study are those of CF_0.71_ ((C_2_F)_*n*_ + (CF)_*n*_) and CF_0.51_ ((C_2_F)_*n*_ + (CF)_*n*_)). Our measurements show that it is preferable to keep the graphitic phase rather than (CF)_*n*_ for reflector design, despite the lower fluorination rate. In other words, the content of the (CF)_*n*_ phase must be minimized. Both the location (core-shell or homogeneous) and the size of the residual graphitic phases must be optimized in order to enhance the reflection. We plan to produce a few samples of intercalated graphites with the optimal parameters estimated in this work and to measure the double-differential neutron diffraction cross section in the complete range of wavelengths corresponding to the so-called “reflectivity gap”. This measurement will require the construction of a specialized diffractometer on the PF1B instrument, and a sufficiently long measurement time due to the fact that the incident neutron flux decreases rapidly with increasing neutron wavelength. The physicochemical properties of these samples, including their resistance to radiation fluxes, will also be studied in detail. This study is important for determining the conditions under which such reflectors can be used in practice.

## Figures and Tables

**Table 1:** Parameters of the prepared samples. The density of the samples and the sample thicknesses are measured with the accuracy of a few percents.

Sample Name	Density, g · cm^−3^	Thickness, cm	F/C Ratio	${\left(C_{2}F\right)}_{n}$ Fraction, %	Interlayer Distance, Å	Grain Size, µm
ARC3	0.85	1.2	1.01	0	6.2	1–45
ARC4	0.83	1.2	0.87	0	6.2	1–45
ARC5	0.84	0.8	0.63	0	6.2	1–45
ARC6	0.81	1.2	0.37	0	6.2	1–45
ARC7	0.85	1.2	0.18	0	–	1–45
ARC8	0.87	0.8	0.01	0	–	1–45
CF_0.51_	0.35	1.2	0.51	51	8.9	7.5
CF_0.61_	0.42	0.8	0.61	61	8.9	7.5
_0.71_	0.38	0.8	0.71	45	8.0	7.5
FKS450	0.83	0.8	0.57	74	9.0	44
FNG440	0.35	1.2	0.52	94	9.2	200–400
FNG460	0.84	1.2	0.55	96	9.2	200–400
FNG600	0.76	0.8	0.60	67	9.2	200–400
RT-GF	0.43	0.8	0.71	0	6.0	7.5

## Data Availability

Neutron data were obtained from experiment TEST-3370 of the PF1B instrument at the ILL, Grenoble, France.
